# Case Report: The Cox-Maze IV Procedure in the Mirror: The Use of Three-Dimensional Printing for Pre-operative Planning in a Patient With Situs Inversus Dextrocardia

**DOI:** 10.3389/fcvm.2021.722413

**Published:** 2021-09-14

**Authors:** Long Song, Chengming Fan, Hao Zhang, Hongduan Liu, Chukwuemeka Daniel Iroegbu, Cheng Luo, Liming Liu

**Affiliations:** Department of Cardiovascular Surgery, The Second Xiangya Hospital, Central South University, Changsha, China

**Keywords:** Cox-maze IV procedure, mirror-image dextrocardia, three-dimensional printing, pre-operative planning, surgery

## Abstract

The safety and efficacy of the Cox-Maze IV procedure (CMP-IV) for situs inversus dextrocardia patients with atrial fibrillation is yet to be determined. Herein, we present the case of a 39-year-old male patient admitted to our cardiac center following progressive exertional dyspnea. The patient was diagnosed with situs inversus dextrocardia, severe mitral regurgitation, and paroxysmal atrial fibrillation. A three-dimensional (3D) heart model printing device embedded with designated ablation lines was used for pre-operative planning. Mitral valvuloplasty, CMP-IV, and tricuspid annuloplasty were performed. The patient had an uneventful recovery and was in sinus rhythm during a 12-month follow-up period using a 24-h Holter monitoring device. The case herein is one of the first to report on adopting the CMP-IV procedure for situs inversus dextrocardia patients with complex valvuloplasty operation. In addition, the 3D printing technique enabled us to practice the Cox-maze IV procedure, given the patient's unique cardiac anatomy.

## Background

The Cox-Maze IV procedure (CMP-IV) is the only operation and technology with an FDA-approved indication for the surgical treatment of AF. However, the safety and efficacy of the CMP-IV for situs inversus dextrocardia patients with atrial fibrillation is yet to be determined. The case herein is one of the first to report on adopting the CMP-IV procedure for mirror-image dextrocardia patients with complex valvuloplasty operation with the guidance of the 3D printing technique.

## Case Presentation

**Figure 1:**
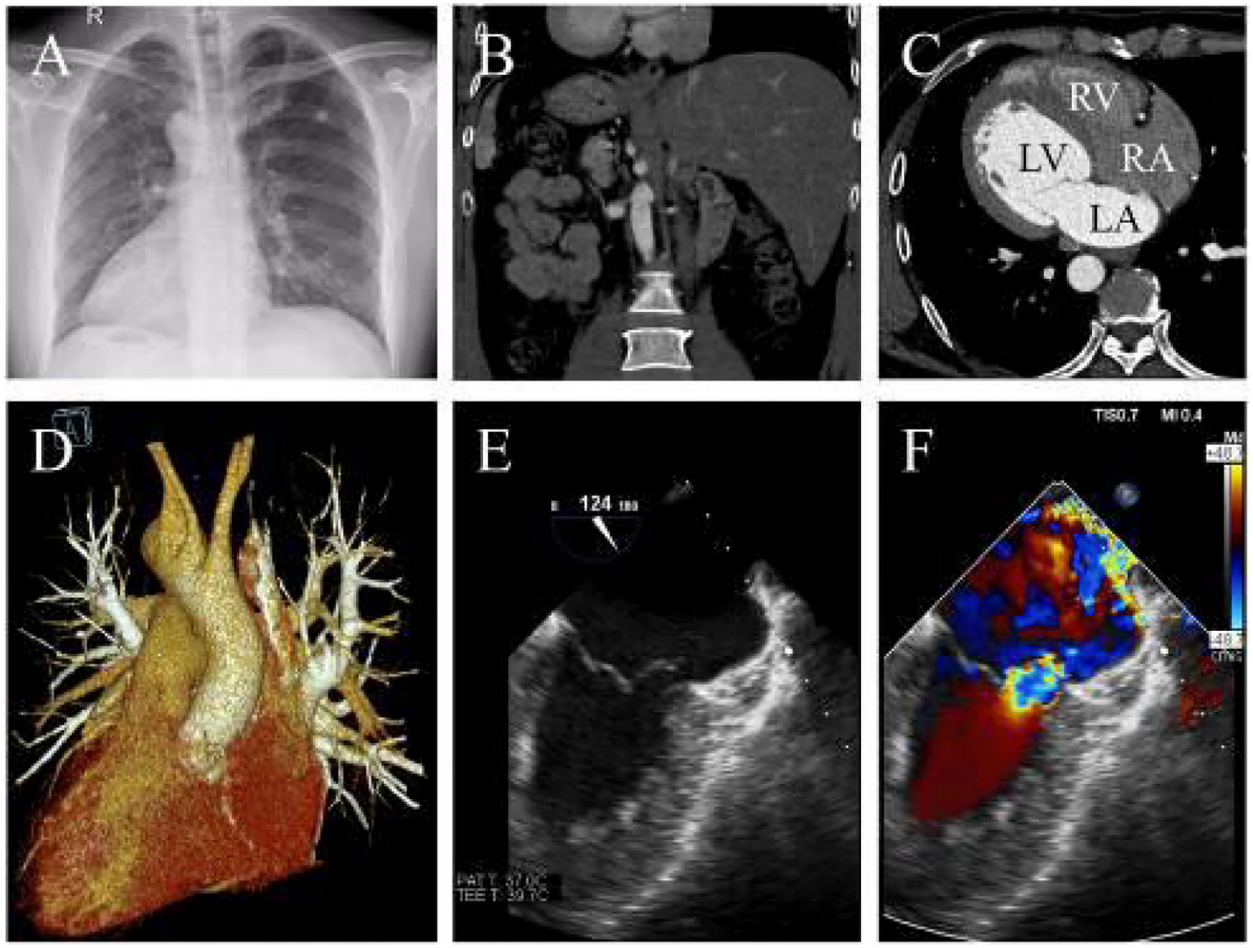
**(A)** X-ray indicates dextrocardia and cardiac enlargement. **(B)** CTA reveals transposition of the abdominal organs and **(C)** confirming L-loop ventricular orientation and concordant atrioventricular connections. **(D)** Three-dimensional CTA shows normal great artery development and connections. **(E)** The transesophageal echocardiography shows mitral chordae rupture and **(F)** severe mitral regurgitation (LA, left atrium; LV, left ventricle; RA, right atrium; RV, right ventricle).

A 39-year-old man who complained of progressive exertional dyspnea and intermittent palpitation was referred to our department. He denied any familiar history of situs inversus dextrocardia or other cardiac health comorbidities. The patient, however, had occasional dizziness but denied any history of hypertension or cerebral infarction. Physical examination revealed a systolic blowing murmur (grade 3/6) at the fifth intercostal space lateral to the right midclavicular line. Chest roentgenogram and computed tomography (CT) showed dextrocardia with an enlarged silhouette and situs inversus totalis (Figures 1A,B). The anatomic diagnosis was mirror-image dextrocardia, L-loop ventricles, and typical related great arteries without associated congenital cardiac abnormalities (Figures 1C,D). Transesophageal echocardiography further revealed severe mitral valve regurgitation where the vena contracta was >0.7 cm and tricuspid regurgitation following annular dilation. The left atrium diameter was 5.8 cm without thrombosis formation (Figures 1E,F and Supplementary Video 1). A 24-h Holter monitoring demonstrated paroxysmal AF with a total burden of 165 min.

**Figure 2:**
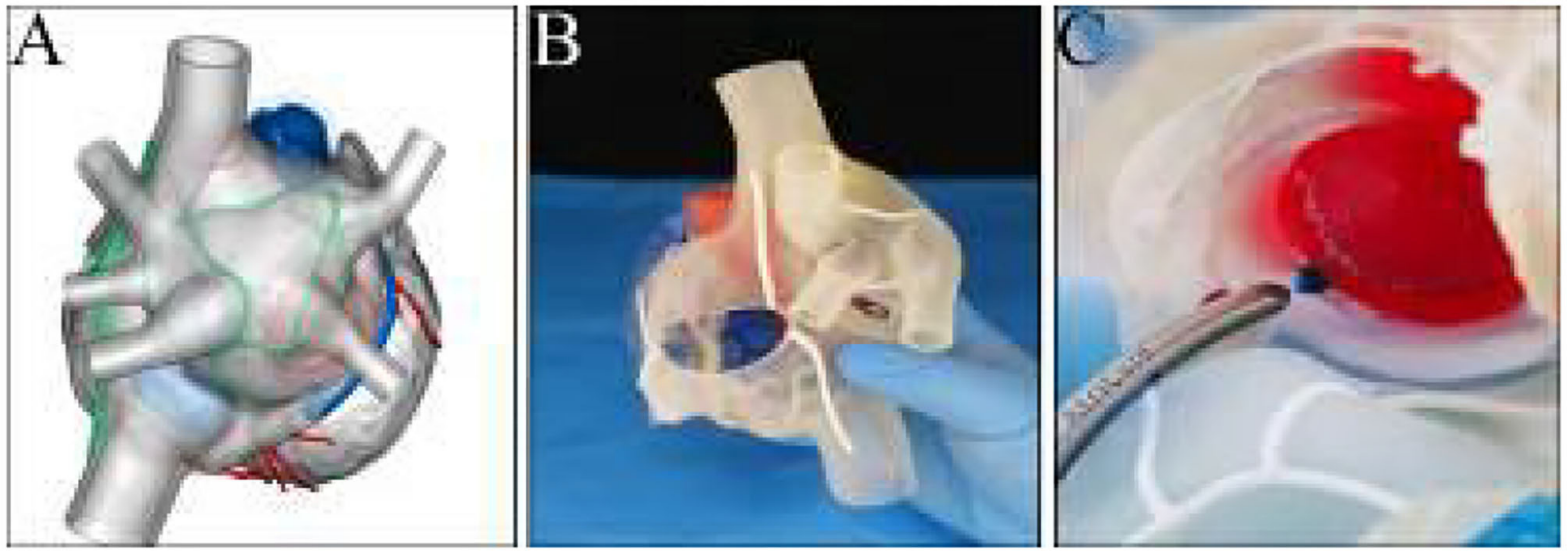
**(A)** Pre-printing digital file of the 3D model embedded with designated ablation lines. **(B)** View of the three-dimensional printed model. **(C)** Clamping on the model of the mitral isthmus line during the rehearsal process.

Three-dimensional (3D) printing of the heart was performed using the derived cardiac CT data to precisely comprehend the anatomy and guide surgical ablations. A stereolithography file of the 3D model embedded with designated ablation lines was generated (Figure 2A), which was then manufactured with soft and flexible resinous material at a ratio of 1:1 (Figure 2B). The easy-to-cut and retractable feature of the 3D model (Supplementary Video 2) allowed us to rehearse the CMP-IV pre-operatively (Figure 2C).

**Figure 3:**
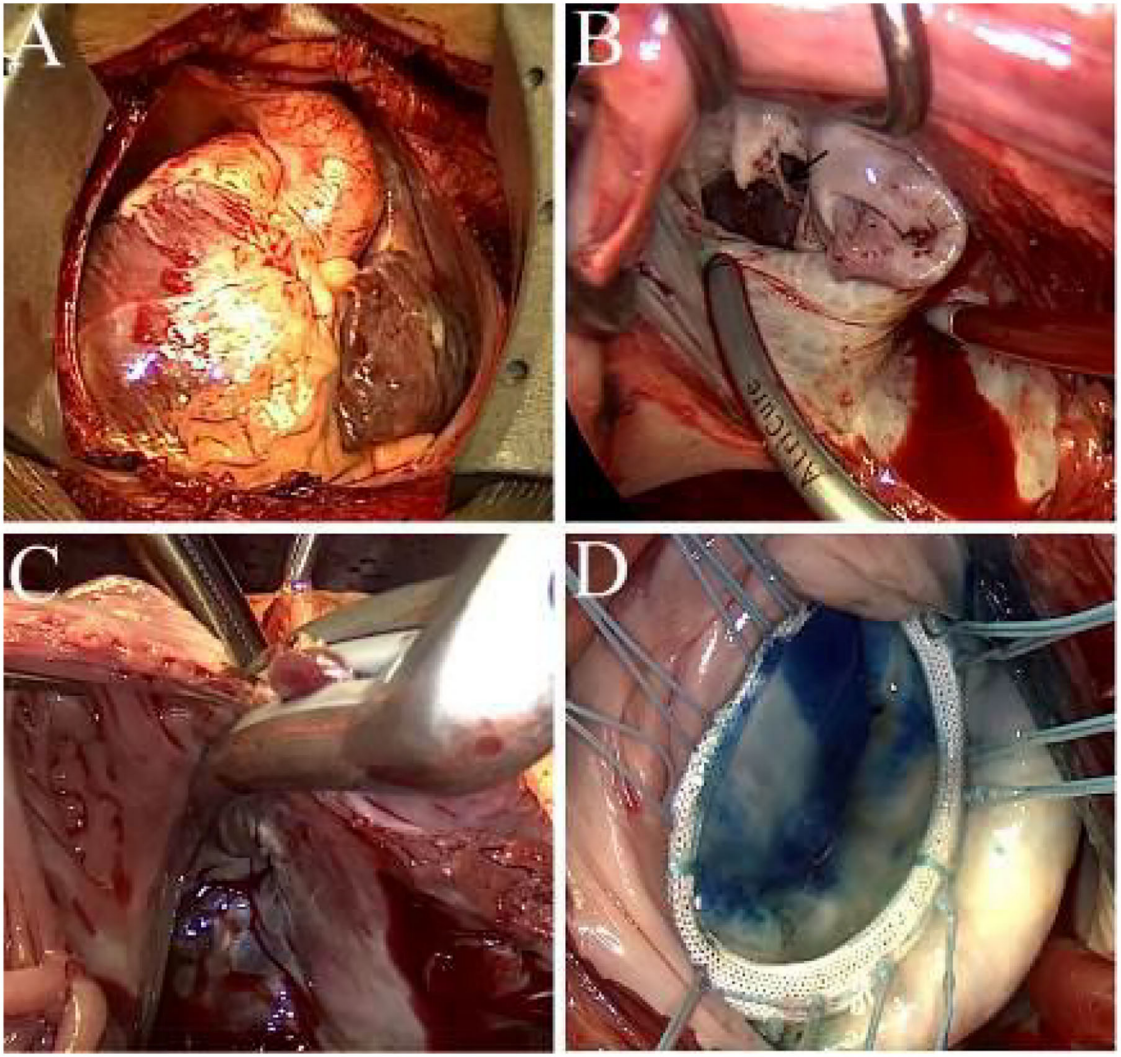
**(A)** Intraoperative view of the mirror-image dextrocardia. **(B)** Ablation at the mitral line, black arrow indicates the ruptured chordae. **(C)** Ablation to the tricuspid annulus, the jaw of the bipolar clamps are placed across the tricuspid annulus. **(D)** Successful result of saline injection test after mitral valvuloplasty.

The cardiopulmonary bypass for the patient was routinely performed via a median sternotomy (Figure 3A). The operator operated on the patient's left side while the cardiopulmonary bypass machine was placed on the right side. A biatrial CMP-IV procedure with mitral valvuloplasty and tricuspid annuloplasty were simultaneously performed (Supplementary Video 3). The left atrium was accessed via the interatrial groove. We lengthened the mitral isthmus lesion to the posterior mitral valve annulus for the left atrial lesion sets and ablated the coronary sinus endocardially and epicardially with a bipolar radiofrequency pen (AtriCure Inc., Cincinnati, OH). Other lesions, which were created using bipolar radiofrequency clamps (AtriCure Inc., Cincinnati, OH), included: (i) bilateral pulmonary veins isolation; (ii) ablation lines connecting the left atrial appendage and the left superior pulmonary vein; (iii) ablation lines connecting the right and the left superior pulmonary veins; (iv) ablation lines connecting the right and the left inferior pulmonary veins, and (v) mitral line lesions (Figure 3B). Finally, the ligament of Marshall was dissected, and the left atrial appendage was isolated using an epicardial AtriClip closure device. The entire right atrial lesion sets were created using bipolar radiofrequency clamps, including the annular tricuspid lesions (Figure 3C), superior and inferior vena cava lesion lines, and lesion lines connecting the right atrium incision to the right atrial appendage.

We performed A1 ruptured chordae resection, followed by implantation of a single flexible artificial chord with 4-0 Gore-tex *in situ*. The residual leak of the anterior commissure and the A2 leaflet cleft was closed accordingly. A 32 mm rigid ring (Element Force ARM32, Kingstron Bio, Suzhou, China) was then implanted to stabilize the mitral annulus. The coaptation height was 9 mm after reconstruction of the mitral valve (Figure 3D). After the closure of the interatrial groove incision and de-airing, the aortic clamp was removed, and the right atrium was entered via a longitudinal incision.

The tricuspid repair was obtained with a 28 mm band (SOVERING TRICUSPID BAND^TM^, Sorin Group Italia Srl, Italy) due to the mirror-imaged anatomy. The patient was in sinus rhythm before cardiopulmonary bypass weaning, and no atrioventricular block was detected. He had an uneventful recovery, and the anti-arrhythmia and oral anticoagulation drugs were continued for 3-months post-operatively. The patient was in sinus rhythm (24-h Holter monitoring device) with an improved quality of life during a 12-month follow-up period.

## Discussion and Conclusions

Mirror-image dextrocardia is characterized by a mirror-image change of the normal heart mostly accompanied by situs inversus viscerum with only 3–10% intracardiac anomaly (1). The CMP-IV procedure is the only operation and technology with an FDA-approved indication for the surgical treatment of AF. The term “Maze” is appropriately used only to refer to the biatrial lesion sets of the CMP-IV. Less extensive lesion sets should not be referred to as the CMP-IV but rather as a surgical ablation (2).

Studies have confirmed that the Cox-maze IV procedure concomitant with valvular surgery had higher efficiency concerning AF elimination, AF burden relief, stroke rate control, long term survival rate, and health-related quality of life improvement than valvular surgery alone, catheter ablation, and other forms of surgical AF ablation (3–5). CMP-IV showed excellent efficacy in restoring sinus rhythm during the 10-years follow-up in the studies mentioned above. The procedure was performed in patients with paroxysmal and non-paroxysmal AF (6). However, age, AF duration, non-paroxysmal AF, and left atrial size were adverse prognostic factors (6, 7) for the long-term outcome of the Cox-Maze IV procedure. Surgical ablation experiences for AF patients with dextrocardia have been sporadically reported (8). Due to the low incidence rate, there is a paucity of data describing the safety and efficacy of CMP-IV *in situs* inversus dextrocardia patients, let alone its long-term outcomes and durability.

The ablation device used herein was the bipolar radiofrequency clamps combined with a bipolar radiofrequency pen, given cryothermal energy is not available in China. We performed bipolar radiofrequency clamping in three sets for each lesion line with two applications per set without unclamping, which has been documented to result in 100% atrial lesion transmurality (9). A complete set of right atrial lines was performed with bipolar radiofrequency (10). Dissection of the epicardial fat of the right atrioventricular groove not only warranted tricuspid annular clamping but also avoided right coronary artery injury. Incomplete ablation of mitral isthmus had been reported to cause post-operative atrial flutters (11). The painting and stamping ablation technique was used for mitral isthmus lesions with a bipolar radiofrequency pen given the location. No post-operative atypical atrial flutter was detected.

The pulmonary veins are a crucial source of ectopic beats, triggering frequent paroxysmal AF (12). However, the duration of AF directly influenced the degree of atrial remodeling and the complexity of the atrial substrate (13). Though the patient had paroxysmal AF, we decided to apply the CMP-IV procedure rather than the modified left atrial lesion set or a more straightforward pulmonary vein isolation. The decision was based on the fact that patients with extended paroxysmal AF may have an advanced underlying disease than realized, especially if there are signs of left atrial enlargement (14).

Currently, there is a knowledge-practice gap between Class-1 recommendation on concomitant surgical ablation for AF (2) and its low adoption rate (15). One of the barriers impeding the practice was the unfamiliar cardiac anatomy, which made the procedure challenging, extending the cardiopulmonary bypass and aortic cross-clamp time. The other stumbling block was that no standardized curriculum exists for training cardiothoracic surgery residents in surgical ablation for AF.

Exposure to complete ablations and familiarization with the anatomic boundaries may be accomplished in the way of simulation, tissue labs, or higher-fidelity models (16). In the case herein, the 3D printing model enabled a dynamic visualization of the spatial relationship between the specific ablation lines and the critical anatomic references such as the coronary sinus, the mitral isthmus, the posterior mitral annulus, and the right coronary artery. In addition, the rehearsal process provided an intuitive perspective concerning viable ablation strategies, including the location, direction, and length for each clamping to ensure continuous lesion sets and avoid potential damages to the surrounding structures.

**Figure 4:**
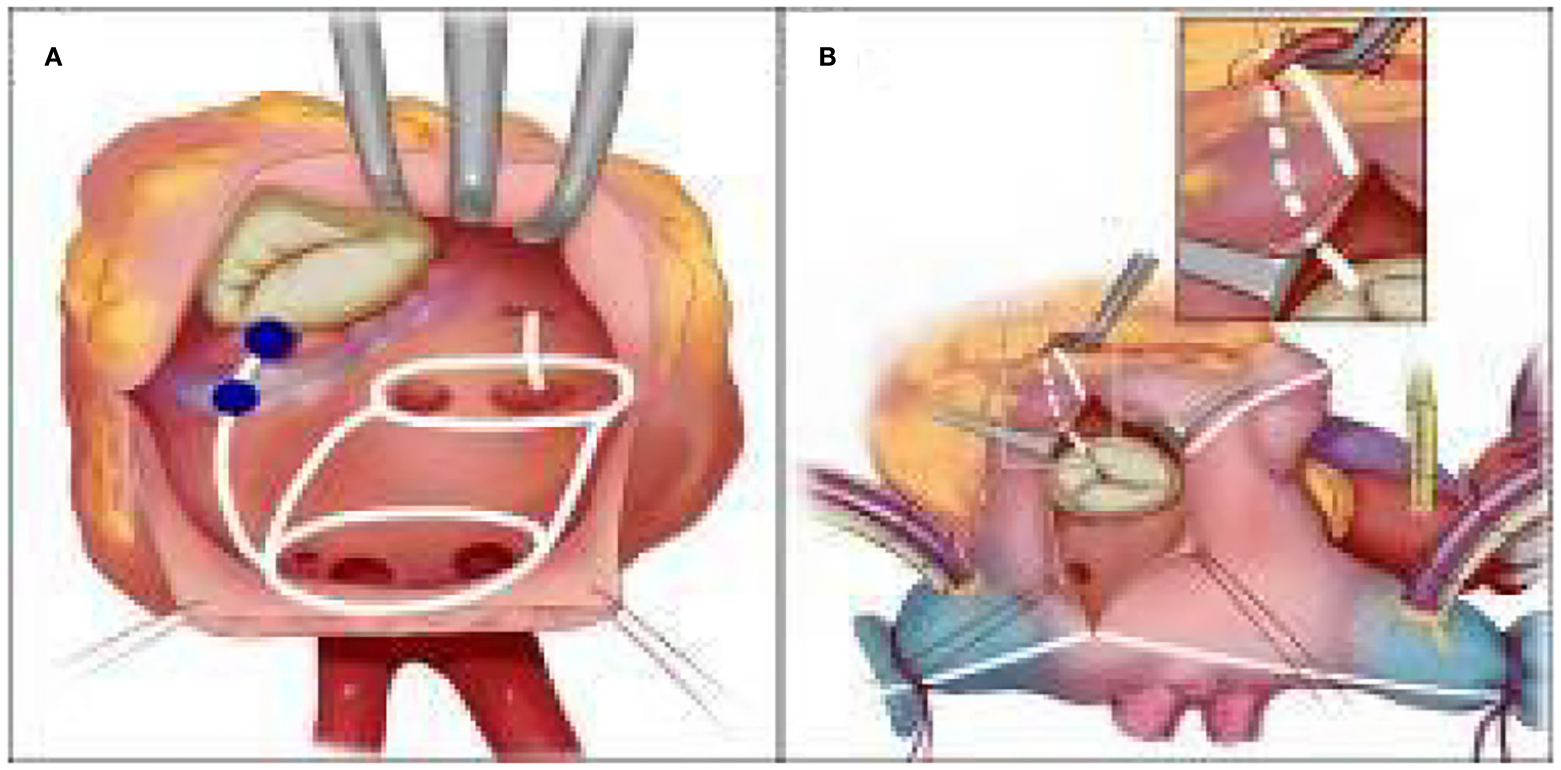
**(A)** Left atrial lesion sets for Cox-maze IV procedure in the mirror-image dextrocardia, Mitral isthmus lesion is between the shaded color (light and dark blue) at the posterior mitral annulus and the coronary sinus. **(B)** Right atrial lesion sets for Cox-maze IV procedure in the mirror-image dextrocardia. The tricuspid annulus lesion is completed after dissecting the peripheral epicardial fat of the right coronary artery.

The Cox-Maze IV procedure in such a rare malformation was complex and technically challenging. We believe that dedicated pre-operative ablative lesion sets (Figure 4) and procedural rehearsal with 3D printing heart models are of added value given that it shortens operation time and increases the chance to achieve better results than the empirical “track in mind” does. Our individualized 3D printing model embedded with designated lesion lines may also serve as a potential candidate for clinical instruction in AF surgery.

Nonetheless, the study herein is without its limitations. The study is a single-patient case report, given the low incidence rate and the rarity of the disease. Our 3D printing heart model could not be used for a pre-operative mitral or tricuspid rehearsal valve repair. After the procedure, the lack of electroanatomical mapping is the only test to verify if the ablation lines were adequately performed and the desired block reached. However, the latest guidelines (2, 17) do not recommend routine intraoperative mapping for each lesion line of the Cox-maze IV procedure to prove if the derived electrophysiology was effective. We thus strongly believe that an in-depth collaboration between the cardiac surgeon and the electrophysiologist would widen the spectrum for the effective treatment of valvular AF in the future.

To our knowledge, the study herein is one of the first to adopt the CMP-IV procedure in a patient with situs inversus dextrocardia, which was successfully guided using rapid-prototyping techniques for the preparation of individualized lesion sets (18). In addition, the 3D printing model helps surgeons mimic and modify the CMP-IV procedure in patients with unfamiliar cardiac anatomy, particularly with rare malformations, supporting pre-operative planning and training (19) in surgical ablation for AF.

## Data Availability Statement

The original contributions presented in the study are included in the article/Supplementary Material, further inquiries can be directed to the corresponding authors.

## Ethics Statement

The studies involving human participants were reviewed and approved by the Ethics Committee of the Second Xiangya Hospital of Central South University. The patients/participants provided their written informed consent to participate in this study. Written informed consent was obtained from the patient for publication of this case report and any accompanying images.

## Author Contributions

LS and CF drafted the manuscript. LL designed the study. HZ, HL, CI, CL, and CF revised the manuscript. LS, HZ, and HL were responsible for the collection of data or analysis. All authors read and approved the final manuscript.

## Funding

This work was supported by the National Key Research and Development Program (Grant No. 2018YFC1311204 to LL) and Natural Science Foundation of Hunan Province (Grant No. 2021JJ30951 to LS).

## Conflict of Interest

The authors declare that the research was conducted in the absence of any commercial or financial relationships that could be construed as a potential conflict of interest.

## Publisher's Note

All claims expressed in this article are solely those of the authors and do not necessarily represent those of their affiliated organizations, or those of the publisher, the editors and the reviewers. Any product that may be evaluated in this article, or claim that may be made by its manufacturer, is not guaranteed or endorsed by the publisher.

## Supplementary Material

Supplementary Video 1 Transesophageal echocardiography showed severe mitral regurgitation.

Supplementary Video 2 Demonstration of the material used for 3D printing.

Supplementary Video 3 Descriptions of the complete lesion sets and critical steps for valvuloplasty.
